# Echocardiographic assessment of *Xenopus tropicalis* heart regeneration

**DOI:** 10.1186/s13578-023-00982-z

**Published:** 2023-02-13

**Authors:** Luocheng Lv, Weimin Guo, Wei Guan, Yilin Chen, Ruijin Huang, Ziqiang Yuan, Qin Pu, Shanshan Feng, Xin Zheng, Yanmei Li, Luanjuan Xiao, Hui Zhao, Xufeng Qi, Dongqing Cai

**Affiliations:** 1 Key Laboratory of Regenerative Medicine, Ministry of Education, Jinan University, Guangzhou, 510632 People’s Republic of China; 2 Joint Laboratory for Regenerative Medicine, Chinese University of Hong Kong-Jinan University, Guangzhou, 510632 China; 3 International Base of Collaboration for Science and Technology (JNU), Ministry of Science and Technology, Guangzhou, 510632 Guangdong China; 4 Department of Developmental and Regenerative Biology, Jinan University, Guangzhou, 510632 China; 5 Institute of Anatomy, Department of Neuroanatomy, Medical Faculty, University of Bonn, Bonn, Germany; 6 Cancer Institute of New Jersey, Department of Medical Oncology, Robert Wood Johnson of Medical School, New Brunswick, USA; 7 Stem Cell and Regeneration TRP, School of Biomedical Sciences, The Chinese University of Hong Kong, Shatin, Hong Kong, China

**Keywords:** Cardiac regeneration, *X. tropicalis*, Echocardiography

## Abstract

**Background:**

Recently, it was reported that the adult *X. tropicalis* heart can regenerate in a nearly scar-free manner after injury via apical resection. Thus, a cardiac regeneration model in adult *X. tropicalis* provides a powerful tool for recapitulating a perfect regeneration phenomenon, elucidating the underlying molecular mechanisms of cardiac regeneration in an adult heart, and developing an interventional strategy for the improvement in the regeneration of an adult heart, which may be more applicable in mammals than in species with a lower degree of evolution. However, a noninvasive and rapid real-time method that can observe and measure the long-term dynamic change in the regenerated heart in living organisms to monitor and assess the regeneration and repair status in this model has not yet been established.

**Results:**

In the present study, the methodology of echocardiographic assessment to characterize the morphology, anatomic structure and cardiac function of injured *X. tropicalis* hearts established by apex resection was established. The findings of this study demonstrated for the first time that small animal echocardiographic analysis can be used to assess the regeneration of *X. tropicalis* damaged heart in a scar-free perfect regeneration or nonperfect regeneration with adhesion manner via recovery of morphology and cardiac function.

**Conclusions:**

Small animal echocardiography is a reliable, noninvasive and rapid real-time method for observing and assessing the long-term dynamic changes in the regeneration of injured *X. tropicalis* hearts.

**Supplementary Information:**

The online version contains supplementary material available at 10.1186/s13578-023-00982-z.

## Background

Adult injured mammalian cardiac cells have a very limited ability for cell proliferation and regeneration. Today, regeneration of the injured myocardium is still a great clinical challenge. *Xenopus tropicalis* (*X. tropicalis*), a true diploid with high synteny with the human genome [1], has been developed as a useful animal model for developmental and regenerative studies [2–6]. Recently, we established an adult *X. tropicalis* heart apex resection injury model and reported that the adult *X. tropicalis* heart can regenerate in a nearly scar-free manner after injury via apical resection [7]. More recently, we further revealed that cardiomyocyte proliferation plays a critical role in heart regeneration in adult *X. tropicalis*. Fosl1 interacts with JunB to promote the expression of Ccnt1 and then plays a key role in cardiomyocyte proliferation and injured heart regeneration in adult *X. tropicalis* [8]. Compared to the two chambers of the zebrafish heart, *X. tropicalis* has a higher degree of evolution, as the *X. tropicalis* heart has three chambers, with right and left atria and a single ventricle [9]. Thus, a cardiac regeneration model in adult *X. tropicalis* provides a powerful tool for recapitulating a perfect regeneration phenomenon, elucidating the underlying molecular mechanisms of cardiac regeneration in an adult heart, and developing an interventional strategy for the improvement in the regeneration of an adult heart, as the findings from this model may be more applicable in mammals than in species with a lower degree of evolution.

For the study of adult heart regeneration, a noninvasive method that can be used to observe and measure the long-term dynamic changes in the regenerated heart in living organisms is critical to delineate the temporal and spatial sequence of the physiological and pathological occurrences of cardiac regeneration, to develop the temporal and spatial window for effective intervention and to monitor and assess the regeneration and repair status. A recent study reported that echocardiography can assess cardiac morphology and function in *Xenopus* [10]. However, whether the echocardiographic analysis is able to monitor the regeneration of the adult *Xenopus* heart, especially for the assessment of regeneration of injured hearts in scar-free conditions, is still unknown.

In the present study, we applied echocardiography to characterize the morphology, anatomic structure and cardiac function of young injured *X. tropicalis* hearts, which were established by apex resection. The findings of the present study demonstrated for the first time that echocardiographic analysis can assess regeneration of *X. tropicalis* damaged heart in a scar-free perfect regeneration or nonperfect regeneration with adhesion manner via recovery of morphology and cardiac function.

## Materials and methods

### Experimental animals

*X. tropicalis* (Western clawed frogs) at 3–30 months were used for this study. *X. tropicalis* was maintained in a freshwater tank at 26 °C under a 12 h/12 h light/dark cycle. All the experimental protocols related to *X. tropicalis* were approved by the Jinan University Animal Care Committee.

### Anesthesia

Animals were maintained at room temperature (26 to 28 °C) and anesthetized with tricaine mesylate (MS-222; 1 mg/mL; TCI, Shanghai, China). *X. tropicalis* was transferred into a 500 mL bath containing Milli-Q water with 1 mg/mL tricaine mesylate. *X. tropicalis* was identified as being fully anesthetized when there was no movement and no response to touch. This process took approximately 3 to 5 min.

### Echocardiography

**Fig. 1:**
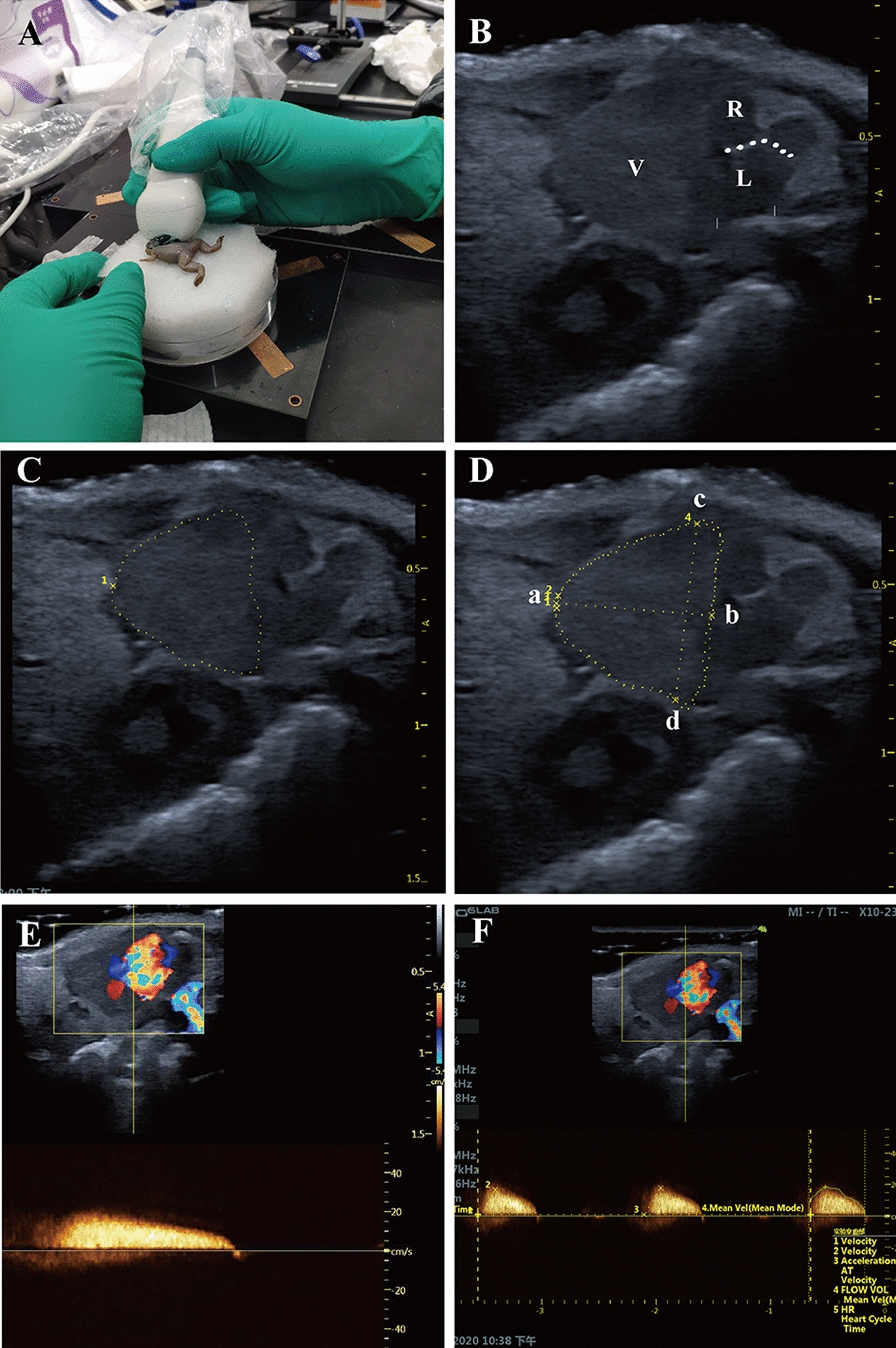
The morphology and anatomy of the *X. tropicalis* heart can be observed clearly using small animal echocardiography **A**: The cardiac images are obtained from the parasternal long-axis of *X. tropicalis* using small animal echocardiography. **B**: Representative image of the subcostal 3-chamber view of the *X. tropicalis* heart under B-mode echocardiography. Ventricle (V). Right atrium (R). Left atrium (L). Atrial septum (dashed line). **C**: Diagram showing the diastole end ventricular girth (dashed line). **D**: Diagram showing the diastole end ventricular length (a to b) and diastole end ventricular width (c to d). **E**: Representative image of pulsed-wave Doppler of the ventricle for hemodynamic analysis. Assessed area (dotted rectangle). **F**: Measurement of peak blood flow velocity and blood flow acceleration of the ventricle. Assessed area (dotted rectangle)

In the present study, small animal echocardiography and pulse Doppler (Vinno 6 Lab, Vinno Technology, Beijing, China) were applied to evaluate the regeneration of *X. tropicalis* under anesthesia. Transducers with broadband frequencies ranging from 10 to 23 MHz (central frequency: 15 MHz), 128 probe elements, a maximum scanning view of 16 mm, and a focal size of 32 mm × 27 mm were used for imaging. The images were collected under B-mode harmonic imaging of the transabdominal ultrasound with the acoustic power, digital gain, frequency, frame rate and dynamic range set as 100%, 45%, 23 MHz, 96.9 Hz and 98 Db, respectively. *X. tropicalis* frogs (3–30 months old) were soaked in tricaine mesylate (MS-222; 1 mg/mL; TCI, Shanghai, China) for 3–5 min. Then, the animals were placed supine on moistened gauze pads to prevent desiccation. The echocardiographic images were collected at the parasternal long axis, as shown in Fig. 1A.

The diastole end ventricular length (mm), diastole end ventricular width (mm), diastole end ventricular girth (mm), diastole end ventricular area (cm^2^), peak blood flow velocity and blood flow acceleration were measured by the Vinno 6 Lab system’s own analysis software. The diastole end ventricular length was set as the distance from the plane point of the atrioventricular valve annulus to the edge of the cardiac apex at the end of diastole, as shown in Fig. 1D (distance from a to b). The diastole end ventricular width was set as the distance between the two uppermost edge points of the end diastolic ventricle, as shown in Fig. 1D (distance from c to d). The diastole end ventricular girth was set as the length of the lateral edge of the end diastolic ventricle (Fig. 1D; yellow dashed line connecting four points, a, b, c, and d), which was measured by the “Perimeter” function. The diastole end ventricular area was set as the area enclosed by the lateral edge line of the ventricle at the end of diastole (Fig. 1D; area surrounded by the yellow dashed line connecting four points a, b, c and d), which was measured by the “Area” function. Because the *Xenopus* ventricle closely approximates an ellipsoid according to optical coherence tomography, ventricular volumes were calculated by using the formula for an ellipsoid model: Volume = 0.85 × (area)^2^/length [11]. Ejection fraction was calculated by using the ventricular volumes: Ejection fraction (%) = [(diastolic volume − systolic volume)/diastolic volume] × 100% [12]. For the comparison of the diastole end ventricular length, diastole end ventricular width, diastole end ventricular girth and diastole end ventricular area, the end diastolic image of each animal as shown in Additional file 1: Fig. S1 (Additional file 1: Fig. S1A for the same age nonapical resection control, Additional file 1: Fig. S1C for the regenerated heart) was applied. For the comparison of the ejection fraction, the end diastolic image and the end systolic image of each animal as shown in Additional file 1: Figure S1 (Additional file 1: Fig. S1B for the same age nonapical resection control, Additional file 1: Fig. S1D for the regenerated heart) were used. The peak blood flow velocity and blood flow acceleration were measured at the most abundant blood flow signals, which were located at the junction of the ventricle and atrium, as shown in Fig. 1E, F. Images of each *X. tropicalis* frog were collected within 5 min after anesthesia. After image acquisition, the animals were put back into the tank for feeding and follow-up observation. In the present study, the criteria for identifying and marking the resected site under echocardiographic image (Fig. 3B, C) is based on the following parameters: lower density and incomplete structure and morphology in the regenerated area combined with the diastole end ventricular length (as shown in a to b of Fig. 1D) of the injured heart is consistent with the length of the heart after removal of the apex.

### Apical resection of the *X. tropicalis* heart

Apical resection of the *X. tropicalis* heart was performed based on our recently established protocol [7]. Briefly, *X. tropicalis* frogs were placed in a tricaine methanesulfonate (MS-222; 1 mg/mL; TCI, Shanghai, China) bath that was prepared with sterile double-distilled water at room temperature for approximately 4 min, incubated on ice for 60 s and then positioned ventral side up on an ice pad. The skin of the chest and upper abdomen was sterilized with iodine and 75% alcohol. A small incision was made near the heart using ophthalmic scissors. The pericardial sac was then opened, and the ventricle was exposed. Approximately 10% (approximately 1 mm in length) of the ventricle tissue from the cardiac apex was resected with Vannas scissors. The opened cavity was sutured with a 4–0 suture after amputation. The animals were subsequently transferred to and maintained in fresh water at 26 °C. The injured hearts were collected at 45 days after apical resection (daar).

### Hematoxylin–eosin staining

The injured hearts collected at different time points were fixed overnight in 4% paraformaldehyde, dehydrated, cleared, and embedded in paraffin wax. Sections with a thickness of 5 μm were prepared for staining. The sections were deparaffinized in xylene (3 × 5 min) and rehydrated with successive 3 min washes in 100%, 100%, 100%, 90%, 80%, and 70% ethanol with one final wash with tap water. The sections were then stained with hematoxylin medium for 15 min, rinsed with tap water for 1 min, rinsed with 1% hydrochloric acid in 80% ethanol for 5 s, rinsed with a 1% ammonia solution for 5 s, and rinsed with tap water for 1 min. The sections were then stained with eosin medium for 3 min and rinsed with tap water for 1 min. After dehydration in an ethanol gradient and clearing with xylene, the slides were mounted with neutral balsam. The stained sections were observed and photographed under a Pannoramic MIDI II scanner system (3DHISTECH, Budapest, Hungary). The images were captured with CaseViewer (version 2.1, 3DHISTECH, Budapest, Hungary).

### Masson’s trichrome staining

Sections were prepared as described above. After deparaffinization and rehydration, the sections were stained with iron hematoxylin for 15 min, rinsed with tap water for 1 min, rinsed with 1% hydrochloric acid in 80% ethanol for 5 s, rinsed with 1% ammonia solution for 5 s, and rinsed with tap water for 1 min. The sections were then stained with Masson’s fuchsin-Ponceau mixture for 25 min and rinsed with tap water for 1 min. Next, the slides were treated for 10 min with phosphotungstic acid orange G and Masson’s light green solution for 4 min. After rinsing with tap water for 1 min, 1% glacial acetic acid was added for 1 min, and the slides were washed with tap water for 1 min. Dehydration in an ethanol gradient and clearing with xylene were conducted, and the slides were mounted with neutral balsam. The stained sections were observed and photographed under a Pannoramic MIDI II scanner system (3DHISTECH, Budapest, Hungary). The images were captured with CaseViewer (version 2.1, 3DHISTECH, Budapest, Hungary).

### Statistical analysis

The results are presented as the mean ± standard deviation (SD). All statistical analyses were conducted using the statistical software SPSS 19.0. One-way ANOVA with the least significant difference (LSD) test was used for intergroup comparisons. A value of *p* < 0.05 was considered statistically significant.

## Results

### The morphology, anatomy and beating of *X. tropicalis* heart can be observed clearly using echocardiography

Young *X. tropicalis* frogs (3 months old) were first applied to test whether the resolution of small animal echocardiograms is appropriate to clearly observe the morphology and anatomy of the heart in a small, 3-month-old frog. *X. tropicalis* was anesthetized with 0.1% tricaine for 3 min. Echocardiography was conducted for *X. tropicalis*. Cardiac images were obtained from the parasternal long-axis. It was found that the image and dynamic beating of the three-chamber view of the *X. tropicalis* heart, which revealed a single ventricle and left and right atria, could reach the resolution required for analysis and could be observed and collected (Fig. 1A–D). In addition, pulsed-wave Doppler for hemodynamic analysis of the ventricle was able to observe and measure the peak blood flow velocity and blood flow acceleration of the ventricle (Fig. 1E, F). The results showed that small animal echocardiography is suitable for analyzing the morphology, hemodynamics and cardiac function of the heart of *X. tropicalis* frogs of 3-months old or older.

### Cardiac measurement of young, young adult and adult *X. tropicalis* hearts using echocardiography

**Fig. 2:**
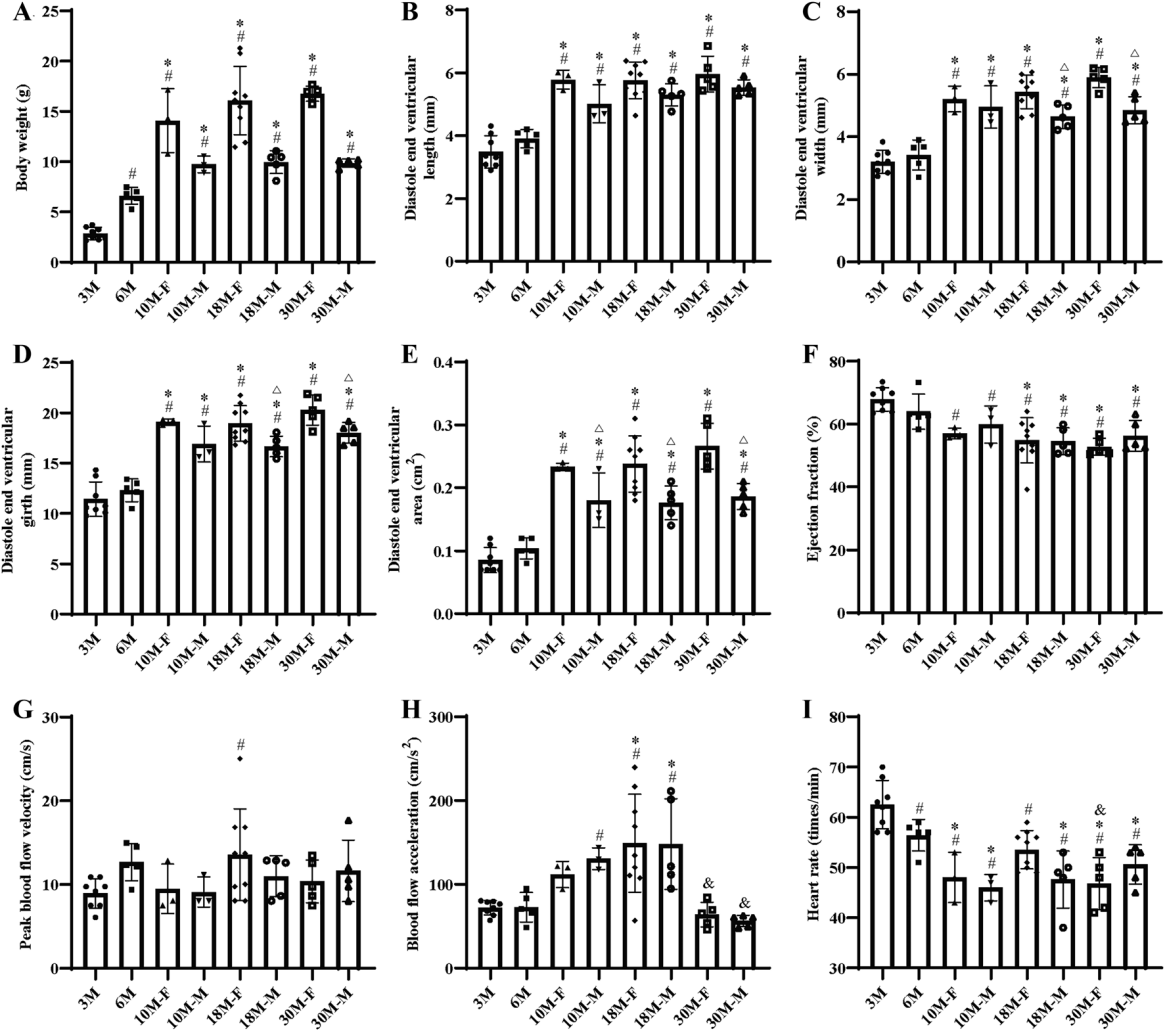
Cardiac measurement of young, young adult and adult *X. tropicalis* Heart using Echocardiography. Body weight **A** in 3-month-old, 6-month-old, 10-month-old, 18-month-old and 30-month-old *X. tropicalis*. Echocardiographic measurement of the diastole end ventricular length **B**, diastole end ventricular width **C**, diastole end ventricular girth **D**, diastole end ventricular area **E**, ejection fraction **F**, peak blood flow velocity **G**, blood flow acceleration **H** and heart rate **I** in 3-month-old, 6-month-old, 10-month-old, 18-month-old and 30-month-old *X. tropicalis* heart. 3 M: 3-month-old. 10 M-F: 10-month-old female. 10 M-M: 10-month-old male. 3 M: n = 8. 6 M, 18 M-M and 30 M-F&M: n = 5. 10 M-F&M: n = 3. 18 M-F: n = 9. #: *p* < 0.05, vs. 3 M. *: *p* < 0.05, vs. 6 M. △:* p* < 0.05, vs. same age female. &:* p* < 0.05, vs. 18 M (same gender and different gender comparison in H, comparison between the same gender in** I**)

Cardiac measurement for young (3-month-old), young adult (6-month-old) and different ages of adult *X. tropicalis* (10-month-old, 18 month-old and 30-month-old) hearts was further investigated by echocardiography. As males and females of *X. tropicalis* at 3 months old and 6 months old cannot be distinguished, no sex group comparison was set for the 3-month-old and 6-month-old groups. The comparison of body weight showed that in male *X. tropicalis*, the body weight increased from 3 to 10 months old, while the body weight increased from 3 to 30 months old in female *X. tropicalis* (Fig. 2A and Additional file 2: Table S1). The results of echocardiography measurements revealed that the mean heart rate was between 46 and 62 (times/minute) in the 3-to 30-month-old age groups in males and females (Fig. 2I and Additional file 2: Table S1). This result suggested that the anesthetic level was similar among the observed age groups and that the possibility of differences in the measured echocardiography parameters due to different anesthesia depths was low. Accordingly, the cardiac measurements of diastole end ventricular length, diastole end ventricular width, diastole end ventricular girth, diastole end ventricular area, ejection fraction, peak blood flow velocity and blood flow acceleration were analyzed. It was found that the mean values of diastole end ventricular length, diastole end ventricular width, diastole end ventricular girth and diastole end ventricular area were increased from 3.49 ± 0.52 mm, 3.21 ± 0.37 mm, 11.41 ± 1.72 mm and 0.09 ± 0.02 cm^2^ to 5.79 ± 0.30 mm (female) and 5.01 ± 0.60 mm (male), 5.21 ± 0.41 mm (female) and 4.95 ± 0.67 mm (male), 19.12 ± 0.28 mm (female) and 16.90 ± 1.78 mm (male) and 0.23 ± 0.01 mm (female) and 0.18 ± 0.04 cm^2^ (male) from 3 months old to 10 months old, while the differences among 10-month-old, 18-month-old and 30-month-old females and males were not statistically significant (Fig. 2B–E and Additional file 2: Table S1). The results suggested that from 3 to 10 months old, the volume of *X. tropicalis* heart is increased. Furthermore, the diastole end ventricular width and the diastole end ventricular girth of female *X. tropicalis* hearts were significantly larger than those of male *X. tropicalis* hearts from 18 months of age onward. However, the diastole end ventricular area of the female *X. tropicalis* heart was significantly larger than that of the male *X. tropicalis* heart from 10 months of age onward (Fig. 2C–E and Additional file 2: Table S1). It is proposed that the volume of the female *X. tropicalis* heart is larger than that of the male *X. tropicalis* heart from 10 months of age onward. In addition, it was found that the ejection fraction (EF) of *X. tropicalis* hearts decreased after 3 months of age and remained at a similar level from 10 to 30 months of age (Fig. 2F and Additional file 2: Table S1). Furthermore, in both males and females, the peak blood flow velocity of *X. tropicalis* hearts was maintained at a similar level from 3 to 30 months old (Fig. 2G and Additional file 2: Table S1), while the blood flow acceleration was increased from 10 months old in males and from 18 months old in females; however, it decreased at 30 months old in both females and males (Fig. 2H and Additional file 2: Table S1). The results suggested that the cardiac function of *X. tropicalis* hearts remained at a relatively stable level from 6 to 30 months of age in both females and males. The findings clearly demonstrated that echocardiographic measurements can be used to monitor and compare the dynamic changes in the morphology, anatomy and cardiac function of *X. tropicalis* hearts from the young to the adult stages.

### Echocardiographic analysis can assess the regeneration of *X. tropicalis* damaged heart in a scar-free manner via recovery of perfect morphology and cardiac function

**Fig. 3:**
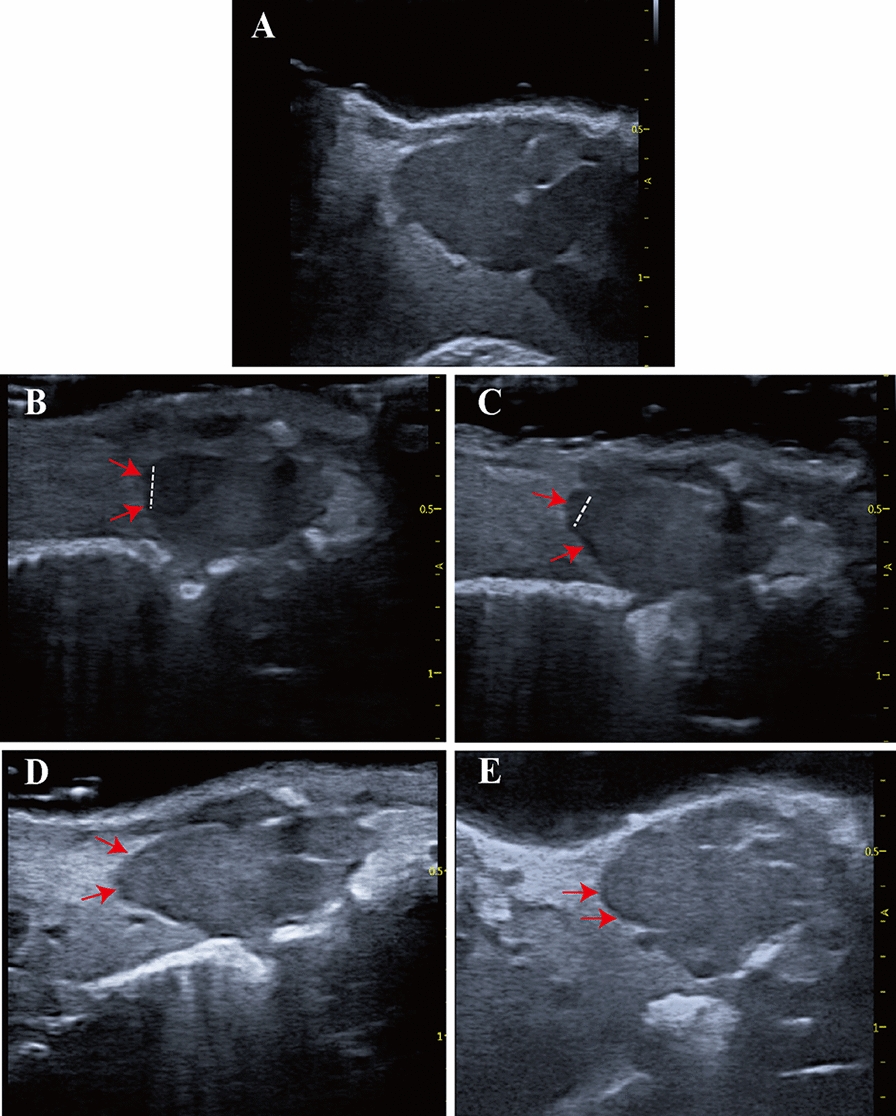
Echocardiographic imaging can monitor regeneration of *X. tropicalis* injured hearts in a scar-free manner. **A**: Representative echocardiography image of the same-age nonapical resection group under B-mode. **B**: Representative image 5 days after apical resection. The damaged heart with a missing apex (left side of the dashed line) was clearly identified under echocardiography. **C**: Representative image 10 days after apical resection. **D**: Representative image 30 days after apical resection. **E**: Representative image 45 days after apical resection. The regeneration of the injured heart was able to be monitored and justified dynamically by the recovery of morphology and anatomic structure under echocardiographic imaging at 5 days, 10 days, 30 days and 45 days after apical resection. The boundary between the apical region of the regenerated heart and the surrounding tissue was clear, and no adhesion with the surrounding tissue was found at 30 days and 45 days after apical resection. Red arrow: Area of the boundary between the apical region of the regenerated heart and the surrounding tissue. Dashed line: Boundary of the regeneration zone and noninjury zone

**Fig. 4:**
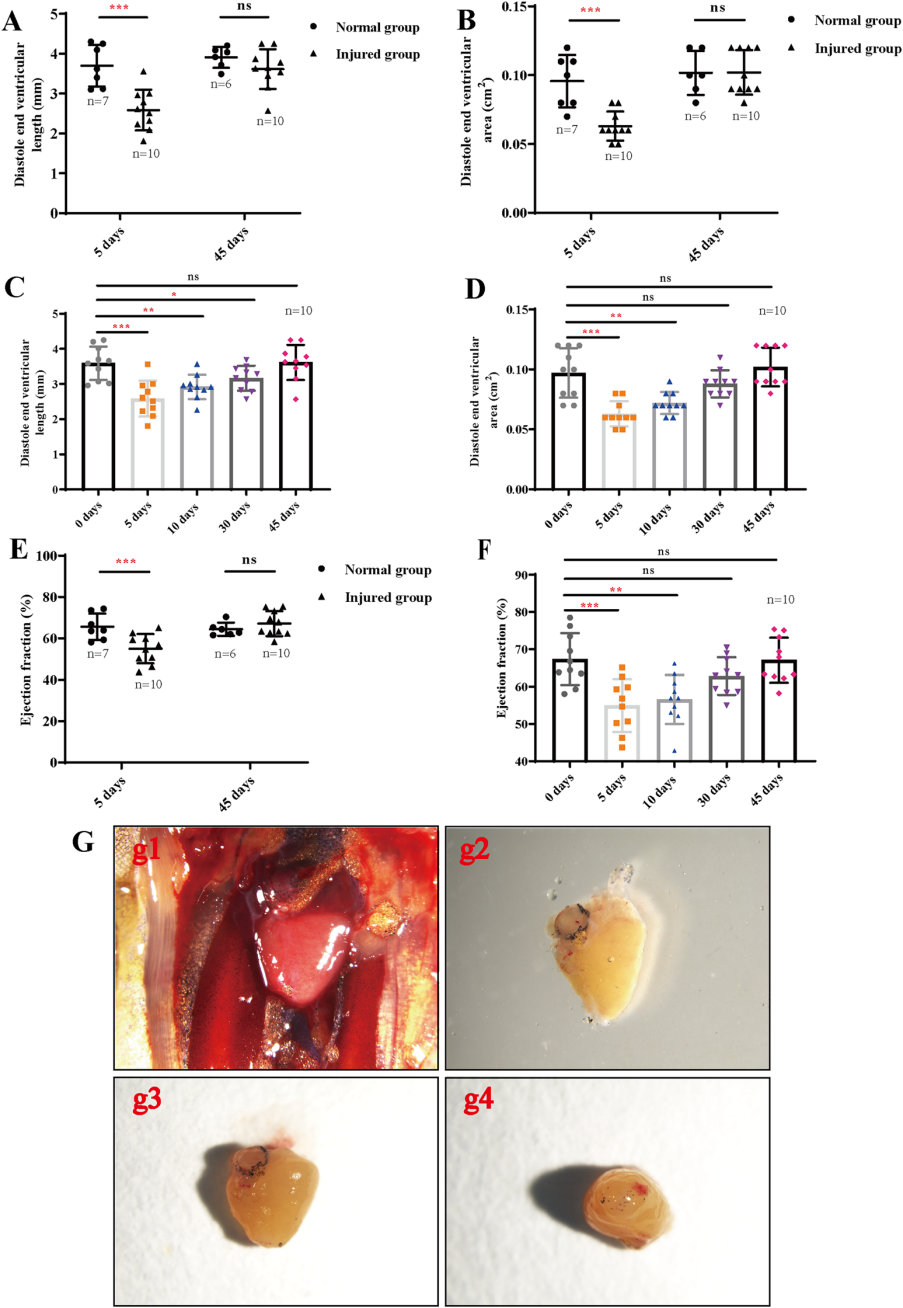
Echocardiographic measurement and ex vivo gross observation confirm regeneration of the *X. tropicalis* injured heart in a scar-free manner. **A** and **B**: The diastole end ventricular length and the diastole end ventricular area of the injured heart group in the 5-daar group were significantly shorter than those of the same-age nonapical resection control group. The differences in the diastole end ventricular length and diastole end ventricular area of the injured heart group in the 45-daar group compared with the same-age nonapical resection control group were not statistically significant. **C** and **D**: The diastole end ventricular length and the diastole end ventricular area of the injured heart group were increased in the 10-daar, 30-daar and 45-daar hearts. At 30 daar, they were close to the same-age nonapical resection control group, while at 45 daar, the diastole end ventricular length and the diastole end ventricular area of the injured heart group were nearly the same compared with the same-age nonapical resection control group. **E**: The ejection fraction (EF) of the injured heart group at 5 daar was decreased significantly compared with the same-age nonapical resection control group. The difference in EF between the injured heart group and the same-age nonapical resection control group at 45 daar was not statistically significant. **F**: The EF of the injured heart was increased at 10 daar, 30 daar and 45 daar. At 30 daar, it was close to the same-age nonapical resection control group, while at 45 daar, the EF of the injured heart group was nearly the same compared with the same-age nonapical resection control group. **G**: Ex vivo gross observation of the regenerated *X. tropicalis* heart at 45 daar, in which echocardiography observation showed that the injured heart was regenerated with no adhesion and was scar-free. G-g1: Representative image of the regenerated *X. tropicalis* heart at 45 daar after in vivo exposure. G-g2: The front side image of the isolated regenerated heart of G-g1. G-g3: Image of the dorsal side of the isolated regenerated heart of G-g1. G-g4: Apical side image of the isolated regenerated heart of G-g1. Ex vivo gross observation confirmed that the cut apex was regenerated with nearly normal morphology, in which the boundary between the apical region of the regenerated heart and the surrounding tissue was clear, and no adhesion with the surrounding tissue was found. 5 daar: 5 days after apical resection

After confirming the feasibility of echocardiography for the analysis of morphology, anatomy and cardiac function of young, young adult and adult *X. tropicalis* hearts, the possibility of echocardiographic analysis to assess the regeneration of *X. tropicalis* injured hearts was further pursued in young *X. tropicalis* hearts. The young heart apical resection injury model was used to establish heart damage as described in our previous report [7]. The echocardiographic analysis for cardiac morphology and measurement was applied 5, 10, 30 and 45 daar. The echocardiographic analysis for the same age nonapical resection control group was set as a control (Fig. 3A). Five days after apical resection (5-daar), the damaged heart with a missing apex was clearly identified under echocardiography (Fig. 3B). The regeneration of the damaged heart was able to be monitored and justified dynamically by the recovery of morphology and anatomic structure under echocardiographic analysis at 5, 10, 30 and 45 daar (Fig. 3B–E vs. A). It was found that at 10 daar, the resected apex had begun to be repaired and presented the morphology of a regenerated apex, which had a lower tissue density than the uninjured area (Fig. 3C). At 30 daar, the regenerated apex became more similar to the normal morphology and anatomic structure, which had a slightly lower tissue density than the noninjured area (Fig. 3D). At 45 daar, the regenerated heart was found to be similar to the normal heart with respect to the morphology, anatomy, tissue density and heartbeat. The boundary between the apical region of the regenerated heart and the surrounding tissue was clear, and no adhesion with the surrounding tissue was found. In addition, when the heart beats, the boundary between the wound area and the surrounding tissue was clear, and no adhesion structure was found to connect with the surrounding tissue (Fig. 3E; Additional file 3: Video S1, Additional file 4: Video S2, Additional file 5: Video S3, Additional file 6: Video S4, Additional file 7: Video S5). In parallel, it was found that the diastole end ventricular length and the diastole end ventricular area of the injured group in the 5-daar group were significantly shorter than those of the same age nonapical resection control group (Fig. 4A, B). Both the diastole end ventricular length and diastole end ventricular area of the injured heart were increased in 10-daar, 30-daar and 45-daar hearts. At 30 daar, they were close to those in the same-age nonapical resection control group, while at 45-daar, the diastole end ventricular length and diastole end ventricular area of the injured heart were nearly the same compared with the same-age nonapical resection control group (Fig. 4C, D). Combined with the echocardiographic morphology findings, these results suggested that the injured hearts were regenerated in a scar-free manner with respect to the morphology and structure. In line with this, the differences in the diastole end ventricular length and diastole end ventricular area of the same-age nonapical resection groups were not statistically significant between 5 and 45 daar (Fig. 4A–D). The results suggest that recovery of the diastole end ventricular length and diastole end ventricular area of the injured heart are attributed to regeneration and not the developmental growth of the damaged heart. In support of this hypothesis, in the injured heart, EF, a parameter of cardiac function, was found to decrease significantly in the 5-daar and then to increase progressively in the 10-daar, 30-daar and 45-daar groups. The EF was close to that of the same-age nonapical resection control group at 30 daar, while at 45 daar, the EF of the injured heart was nearly the same as that of the same-age nonapical resection control group (Fig. 4E, F). Furthermore, the ex vivo gross observation at 45 daar confirmed that the cut apex was regenerated with nearly normal morphology, in which the boundary between the apical region of the regenerated heart and the surrounding tissue was clear, and no adhesion with the surrounding tissue was found (Fig. 4G). Thus, echocardiographic analysis for cardiac measurement and cardiac function can be used to evaluate the dynamics of scar-free regeneration of injured *X. tropicalis* hearts noninvasively in living subjects.

### Echocardiographic analysis can assess adhesion between the regenerated heart and the surrounding tissue to monitor nonperfect regeneration of *X. tropicalis* injured heart

**Fig. 5:**
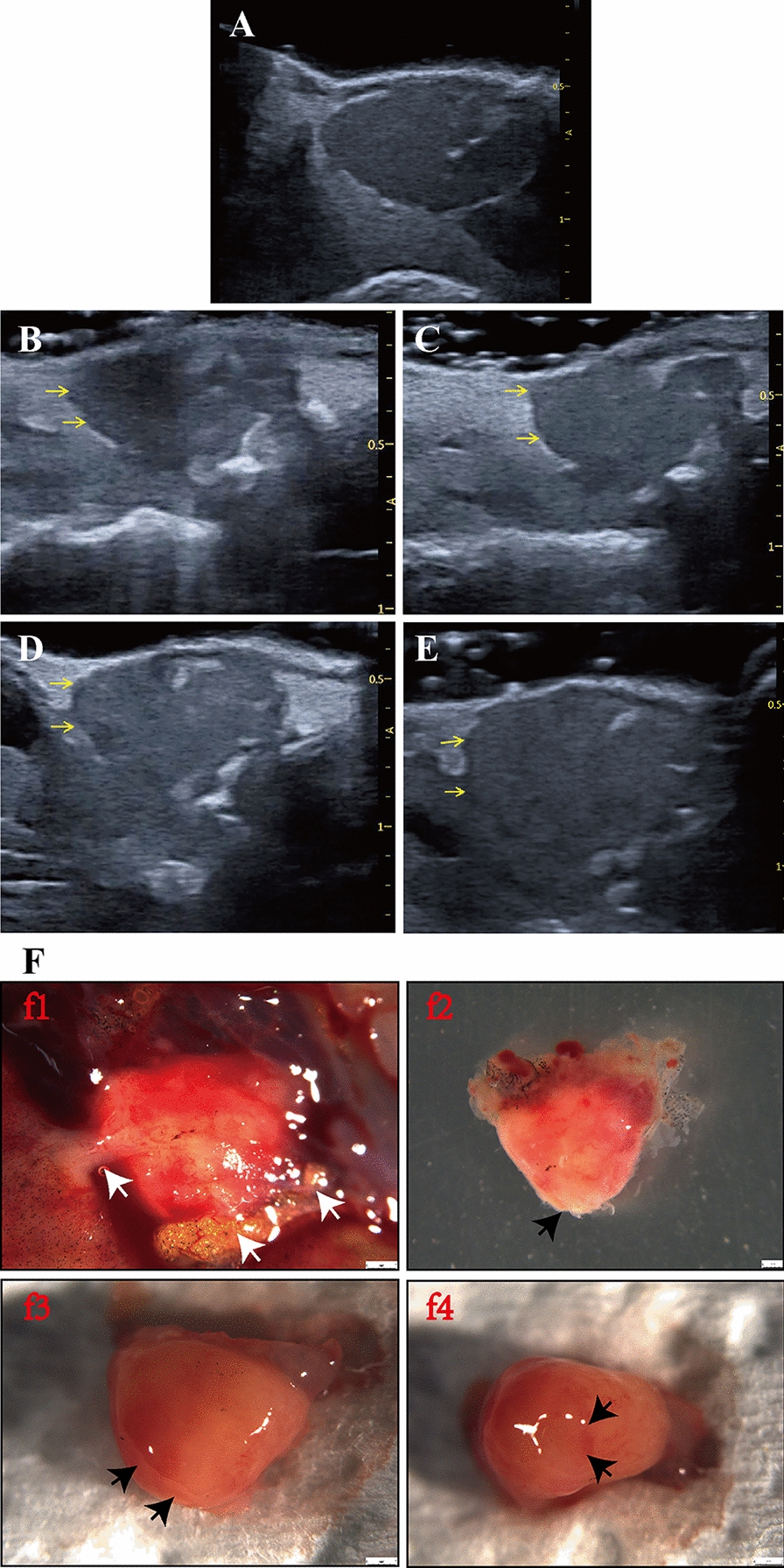
Echocardiographic imaging can monitor nonperfect regeneration with the adhesion of *X. tropicalis* injured hearts. **A**: Representative echocardiography image of the same-age nonapical resection group under B-mode. **B**: Representative echocardiographic image of the injured heart at 5 daar, in which adhesion was identified at the boundary between the apical region of the regenerated heart and the surrounding tissue. **C**: Representative echocardiographic image of the injured heart at 10 daar, in which adhesion was identified at the boundary between the apical region of the regenerated heart and the surrounding tissue. **D**: Representative echocardiographic image of an injured heart at 30 daar, in which adhesion was identified at the boundary between the apical region of the regenerated heart and the surrounding tissue. **E**: Representative echocardiographic image of the injured heart at 45 daar, in which adhesion was identified at the boundary between the apical region of the regenerated heart and the surrounding tissue. Yellow arrow: The adhesion between the apical region of the regenerated heart and the surrounding tissue. **F**: Ex vivo gross observation of the regenerated *X. tropicalis* heart at 45 daar, in which echocardiography observation showed that the injured heart was regenerated with nonperfect regeneration with adhesion. F-f1: Representative image of the regenerated *X. tropicalis* heart at 45 daar after in vivo exposure. White arrow: Adhesion tissue. F-f2: Front side image of the isolated heart of F-f1. F-f3: Image of the dorsal side of the isolated heart of F-f1. F-f4: Apical side image of the isolated heart of F-f1. Black arrow: A defect after the adhesion tissue was cleaned during heart isolation. Ex vivo gross observation confirmed that the cut apex was regenerated in a nonperfect manner with adhesion, in which the boundary between the apical region of the regenerated heart and the surrounding tissue was connected to adhesion tissue. 5 daar: 5 days after apical resection

As in our previous study, it was found that approximately 20% of the adult injured *X. tropicalis* hearts that were established by apical resection could not be regenerated scar-free and experienced adhesion during wound repair [7]. However, the underlying molecular mechanism by which some adult injured *X. tropicalis* hearts fail to regenerate in a scar-free manner is unknown, and it is a very interesting issue to establish an interventional strategy to improve adhesion repair in injured hearts. With this goal, echocardiographic analysis was further investigated to determine whether it could monitor and assess the occurrence of adhesion repair during the regeneration of injured *X. tropicalis* hearts. Indeed, in this study, two of a total of 10 observed injured hearts were found, and the boundary between the wound of the apical region and the surrounding tissue was not identified clearly from 5 daar onward compared to the same-age nonapical resection control group (Fig. 5B vs. A). It was found that when the heart beats, the boundary between the wound area and the surrounding tissue was connected with adhesion tissue (Fig. 5B–E vs. A; Additional file 8: Video S6, Additional file 9: Video S7, Additional file 10: Video S8, Additional file 11: Video S9). Furthermore, the ex vivo gross observation (45 daar) confirmed that the resected apex experienced nonperfect regeneration, in which the boundary between the apical region of the regenerated heart and the surrounding tissue was connected with adhesion tissue, and the resected apical region was not completely regenerated and had defects (Fig. 5F). Thus, echocardiographic analysis for cardiac measurement and cardiac function can be used to evaluate the adhesion and imperfect regeneration of injured *X. tropicalis* hearts dynamically in living organisms.

### Histological analysis confirmed the accuracy of echocardiography assessment for scar-free perfect regeneration and nonperfect regeneration with adhesion of injured *X. tropicalis* hearts

**Fig. 6:**
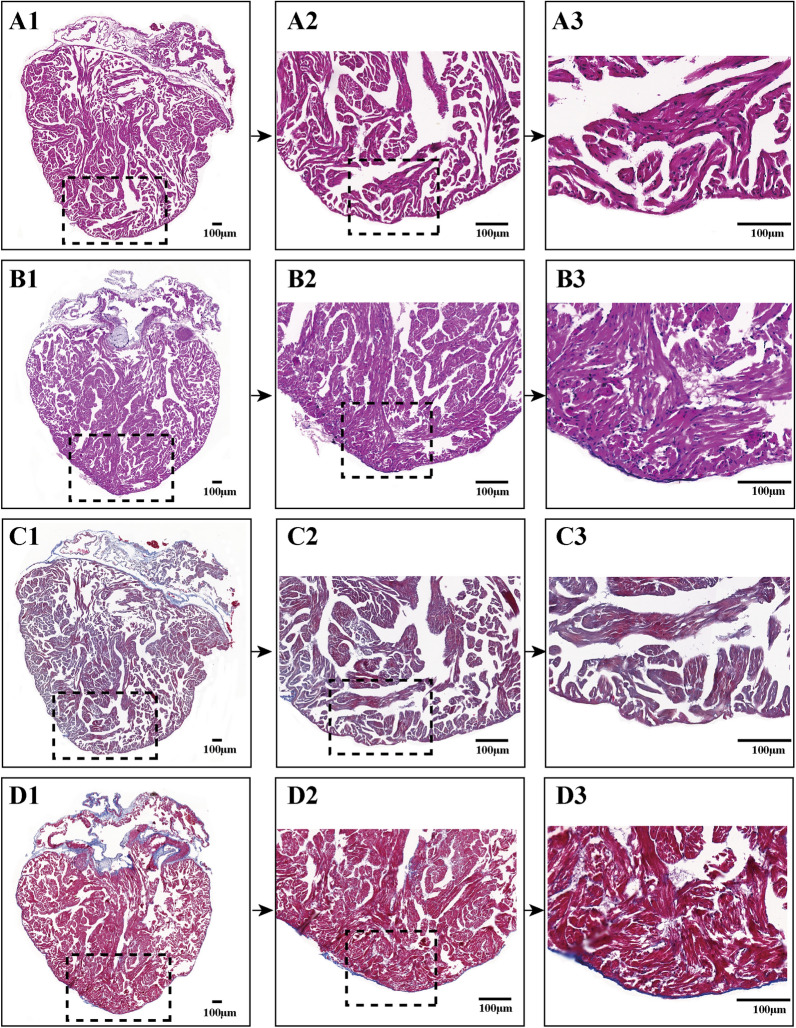
Histology analysis confirms the accuracy of echocardiography assessment for scar-free perfect regeneration of injured *X. tropicalis* hearts.** A1**: Representative H&E staining of the same-age nonapical resection group.** A2**: 3.3 × of dotted rectangle area. **A3**: 2 × of dotted rectangle area.** B1**: Representative H&E staining of the regenerated heart at 45 daar; echocardiography assessment identified regeneration in a scar-free perfect manner.** B2**: 3.3 × of dotted rectangle area. B3: 2 × of dotted rectangle area.** C1**: Representative Masson’s trichrome staining of the same-age nonapical resection group.** C2**: 3.3 × of dotted rectangle area.** C3**: 2 × of dotted rectangle area. **D1**: Representative Masson’s trichrome staining of a regenerated heart at 45 daar; echocardiography assessment identified regeneration in a scar-free perfect manner.** D2**: 3.3 × of dotted rectangle area.** D3**: 2 × of dotted rectangle area. The H&E staining showed that all the amputated areas were replaced by newly regenerated mature cardiomyocytes, as indicated by the regular striations, which were identical to normal control hearts. In addition, the epicardium had regenerated on the outer surface. Furthermore, Masson’s trichrome staining revealed that fibrotic structures were rarely observed in the fully regenerated myocardium and the epicardium, which had regenerated on the outer surface

**Fig. 7:**
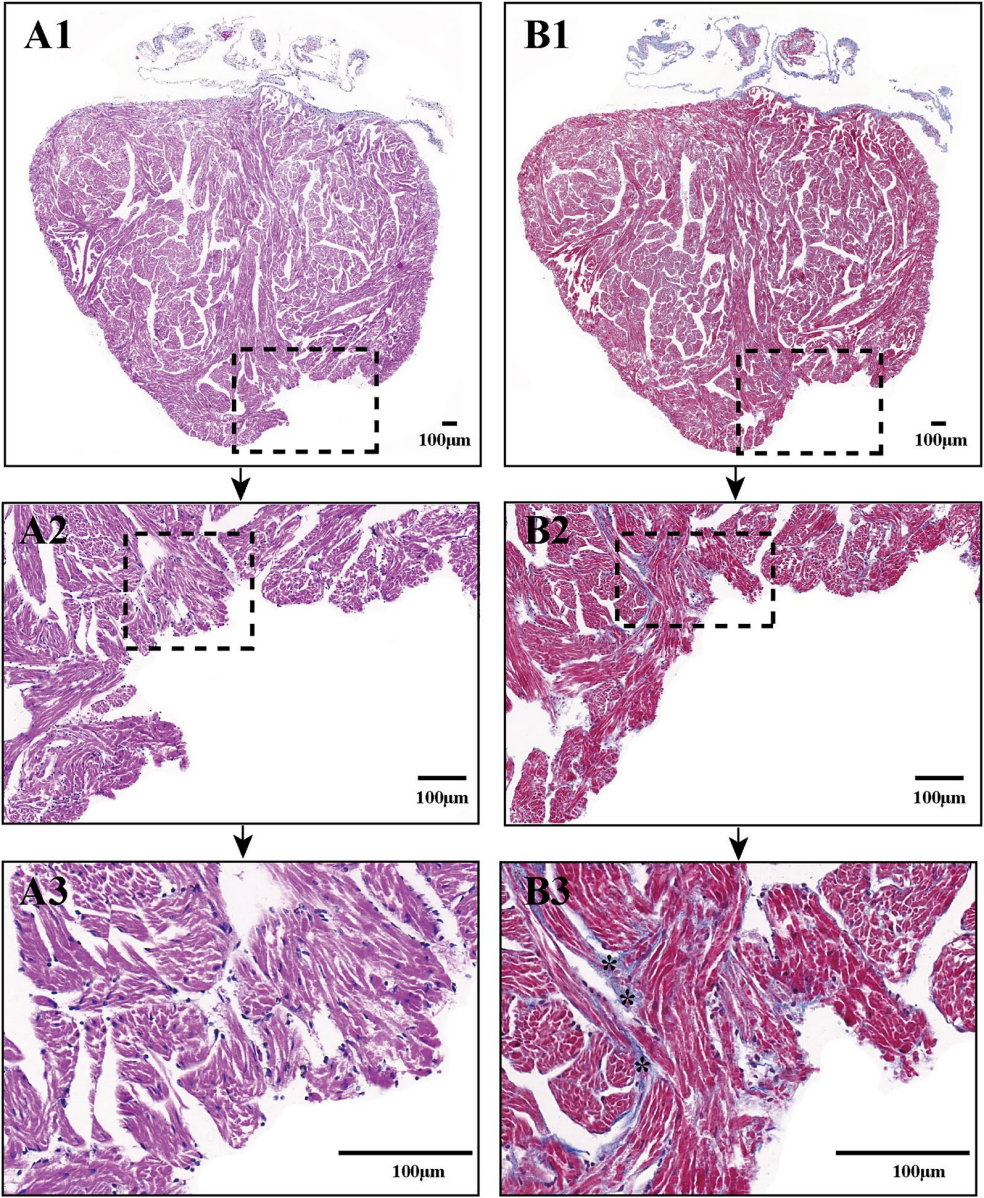
Histology analysis confirms the accuracy of echocardiography assessment for nonperfect regeneration with the adhesion of injured *X. tropicalis* hearts.** A1**: Representative H&E staining of regenerated hearts at 45 daar; echocardiography assessment identified regeneration in a nonperfect manner with adhesion.** A2**: 5 × of dotted rectangle area of** A1**.** A3**: 2.4 × of dotted rectangle area of **A2.**.** B1**: Representative Masson’s trichrome staining of a regenerated heart at 45 daar; echocardiography assessment identified regeneration in a nonperfect manner with adhesion. B2: 5 × of dotted rectangle area of **B1**.** B3**: 2.4 × of dotted rectangle area of** B2**. H&E staining showed that the amputated heart was regenerated with a nearly heart-shaped morphology containing a defect after the adhesion was cleaned. In addition, Masson’s trichrome staining revealed fibrotic structures in the regenerated myocardium. As the adhesion between the regenerated area and peripheral tissue was cleaned when the regenerated hearts were isolated, the fibrotic tissue of the adhesion could not be stained

H&E and Masson’s trichrome staining were applied for histological and fibrosis assessment during the regeneration of injured hearts that were identified as scar-free perfect regeneration by echocardiographic assessment. The results of H&E staining showed that for the regenerated heart (on 45 daar) that was assessed as scar-free perfect regeneration, all of the amputated areas were replaced by newly regenerated mature cardiomyocytes, as indicated by the regular striations that were identical to the same-age nonapical resection control hearts. In addition, the epicardium had regenerated on the outer surface (Fig. 6B1-3 vs. A1-3). In addition, Masson’s trichrome staining of the 45-daar regenerated heart that was assessed as scar-free perfect regeneration showed that, similar to the same-age nonapical resection control heart, fibrotic structures were rarely observed in the fully regenerated myocardium and the epicardium, which had regenerated on the outer surface (Fig. 6D1-3 vs. C1-3). For the regenerated heart (45 daar) that was assessed as nonperfect regeneration with adhesion, the results of H&E staining showed that the amputated heart was regenerated with a nearly heart-shaped morphology containing a defect after the adhesion was cleaned (Fig. 7A). In addition, Masson’s trichrome staining of the regenerated heart showed that a scattered fibrotic structure was observed in the myocardium. As the adhesion tissue between the regenerated area and peripheral tissue was cleaned when the regenerated heart was isolated, the fibrotic tissue of the adhesion could not be stained, and the area was seen as a defect in the regenerated area (Fig. 7B). Thus, the above results of the histological analysis confirm the accuracy of echocardiography assessment for scar-free perfect regeneration and nonperfect regeneration with adhesion for injured *X. tropicalis* hearts.

## Discussion

Today, echocardiography remains a reliable technique for the assessment of cardiovascular structure and function in humans and small animals, such as rodents and zebrafish [13–18]. Ultrasound-based imaging greatly facilitates the monitoring and assessment of cardiac structure and function in genetic modification models (gene knockout and knockin) and surgically induced mouse models [19–21]. However, to date, the methodology of echocardiographic assessment to characterize the morphology, anatomic structure and cardiac function for regeneration of injured adult *X. tropicalis* hearts has not been established. In this study, echocardiographic measurement was applied to characterize the morphology, anatomic structure and cardiac function of young *X. tropicalis* injured hearts, which were established by apex resection to assess regeneration of damaged myocardium in a scar-free perfect regeneration or nonperfect regeneration with adhesion manner via recovery of morphology and cardiac function. We first showed that echocardiographic measurement is feasible to analyze the morphology and cardiac function of *X. tropicalis* hearts that are 3 months old or older. Baseline information of the diastole end ventricular length, diastole end ventricular width, diastole end ventricular girth, diastole end ventricular area, ejection fraction, peak blood flow velocity and blood flow acceleration of young (3-month-old), young adult (6-month-old) and adult (18-month-old and 30-month-old) *X. tropicalis* hearts was investigated and provided to enable scientists to follow late-onset analysis and comparison. The results of echocardiographic measurement documented that from 3 to 10 months old, the volume of the *X. tropicalis* heart is increased, which is supported by age-related increases in the diastole end ventricular length, diastole end ventricular width, diastole end ventricular girth, and diastole end ventricular area. In addition, the volume of the female *X. tropicalis* heart is larger than that of the male *X. tropicalis* heart from 10 months of age onward. Furthermore, it was found that the cardiac function of *X. tropicalis* hearts remained at a relatively stable level from 6 to 30 months old in both females and males, which is supported by the following findings: the ejection fraction (EF) of *X. tropicalis* hearts decreased after 3 months old and were maintained at a similar level from 10 to 30 months old. In both males and females, the peak blood flow velocity of *X. tropicalis* heart was maintained at a similar level from 3 to 30 months old, while the blood flow acceleration was increased beginning at 10 months old in males and at 18 months old in females; however, it decreased at 30 months old in both females and males. The age-related changes in the above parameters clearly suggest that the same-age noninjured control group is necessary for nonbiased comparison when echocardiography is applied to assess the regeneration of injured *X. tropicalis* hearts.

Importantly, the findings of the present study demonstrate that assessment of recovery of the diastole end ventricular length, diastole end ventricular area and EF in *X. tropicalis* heart can assess the regeneration of injured heart with respect to both morphology and cardiac function. Similar to our previous report in which the amputated apex can be regenerated in approximately 30 days [7], in this study, the live echocardiographic imaging showed that at 30 daar, approximately 80% of the regenerated apex of injured hearts (8 hearts) became more similar to normal morphology and anatomic structure, as well as heart beating, wherein the regenerated myocardium still had slightly lower tissue density. At 45 daar, the regenerated heart was found to be similar to the normal heart with respect to morphology, anatomy, tissue density and heartbeat. In addition, no adhesion structure was found between the regenerated apex and the surrounding tissue. In parallel, the quantitative measurements of the diastole end ventricular length, diastole end ventricular area and EF for the regenerated hearts also confirmed that in 30-daar hearts, they were close to the same-age nonapical resection group, while in 45-daar hearts, they were nearly the same compared with the same-age nonapical resection group. Furthermore, the ex vivo gross observation identified that at 45 daar, the cut apex was regenerated with nearly normal morphology, and no adhesion with the surrounding tissue was found. In support of this fact, H&E and Masson’s trichrome staining confirmed histologically that at 45 daar, the amputated area was replaced by newly regenerated mature cardiomyocytes, as indicated by the regular striations, which were identical to the same-age nonapical resection hearts covered with the newly regenerated epicardium, and the amputated area was regenerated in a scar-free manner. All this evidence suggests that echocardiographic analysis can assess regeneration of *X. tropicalis* injured heart in a scar-free manner via recovery of perfect morphology with no adhesion, cardiac measured parameters and cardiac function. Furthermore, our study indicates that the established methodology can quantify the gradual regeneration of injured *X. tropicalis* hearts from assessment of recovery of the diastole end ventricular length, diastole end ventricular area and EF to near normal level within a certain variation range in the suitable time window. Therefore, our established methodology and the parameters can also be applied to analyze the effects of different conditions (such as different time of effectors [growth factors, miRNAs, exosomes], gene alterations, etc.) on the regeneration of *X. tropicalis* heart in a suitable observed time window.

In addition, the findings of the present study also documented that echocardiographic analysis can assess nonperfect regeneration of *X. tropicalis* damaged heart by monitoring the adhesion between the amputated area of the injured heart and the surrounding tissue. Similar to our previous report, in which approximately 20% of injured hearts could not achieve scar-free perfect regeneration but nonperfect regeneration with adhesion [7], in this study, two of 10 total observed injured hearts were found to have a boundary between the wound of the apical region and the surrounding tissue connected with adhesion tissue from 5 daar onward. The ex vivo gross observation combined with H&E and Masson’s trichrome staining confirmed histologically that the cut apex at 45 daar experienced nonperfect regeneration with adhesion. Thus, echocardiographic analysis for cardiac measurement and cardiac function can be used to evaluate the adhesion and imperfect regeneration of injured *X. tropicalis* hearts dynamically in living organisms. Obviously, this feature of echocardiography will be advantageous for investigating the molecular mechanism of scar healing and tailoring a suitable interventional strategy and therapeutic window to improve adhesion repair in injured hearts.

## Conclusions

The established methodology of echocardiographic analysis can assess regeneration of *X. tropicalis* injured hearts in a scar-free perfect regeneration or nonperfect regeneration with adhesion manner via recovery of morphology and cardiac function. We will monitor and assess the regeneration of injured *X. tropicalis* hearts in a reliable noninvasive and rapid real-time manner from the perspective of morphology, structure and cardiac function.

## Supplementary Information

**Additional file 1: Figure S1.** The representative images of the end diastolic stage and the end systolic stage for quantitative comparison of echocardiographic measurement. A: The representative image of the end diastolic level of the same age nonapical resection control heart used for quantitative comparison. B: The representative image of the end systolic level of the same age nonapical resection control heart used for quantitative comparison. C: The representative image of the end diastolic level of the regenerated heart used for quantitative comparison (showing 30 day after apical resection). D: The representative image of the end systolic level of the regenerated heart used for quantitative comparison (showing 30 day after apical resection). The end-diastolic image of each animal was employed for the comparison of the diastole end ventricular length, diastole end ventricular width, diastole end ventricular girth and diastole end ventricular area. For the comparison of the ejection fraction, the end-diastolic image and the end-systolic image of each animal were used.

**Additional file 2: Table S1.** Cardiac measurement data of young, young adult and adult *X. tropicalis *Heart using Echocardiography.

**Additional file 3: Video S1.** Representative echocardiography video of the same-age nonapical resection *X. tropicalis* heart under B-mode.

**Additional file 4: Video S2.** Representative echocardiography video of the injured heart that was regenerated in a scar-free manner 5 days after apical resection under B-mode. The damaged heart with a missing apex was clearly identified under echocardiography.

**Additional file 5: Video S3.** Representative echocardiography video of the injured heart that was regenerated in a scar-free manner 10 days after apical resection under B-mode.

**Additional file 6: Video S4.** Representative echocardiography video of the injured heart that was regenerated in a scar-free manner 30 days after apical resection under B-mode. The boundary between the apical region of the regenerated heart and the surrounding tissue was clear, and no adhesion with the surrounding tissue was found. The regenerated apex became more similar to the normal morphology and anatomic structure but had a slightly lower tissue density than the noninjured area.

**Additional file 7: Video S5.** Representative echocardiography video of the injured heart that was regenerated in a scar-free manner 45 days after apical resection under B-mode. The regenerated heart was found to be similar to the normal heart with respect to morphology, anatomy, tissue density and heartbeat. The boundary between the apical region of the regenerated heart and the surrounding tissue was clear, and no adhesion with the surrounding tissue was found. The regeneration of damaged heart was able to be monitored and justified dynamically by the recovery of morphology and anatomic structure under echocardiographic imaging at 5 days, 10 days, 30 days and 45 days after apical resection (Additional file 4: Video S2, Additional file 5: Video S3, Additional file 6: Video S4, Additional file 7: Video S5 vs. Additional file 3: Video S1).

**Additional file 8: Video S6.** Representative echocardiography video of the injured heart that was repaired in a nonperfect regeneration manner 5 days after apical resection under B-mode. The adhesion was identified in the boundary between the apical region of the regenerated heart and the surrounding tissue.

**Additional file 9: Video S7.** Representative echocardiography video of the injured heart that was repaired in a nonperfect regeneration manner 10 days after apical resection under B-mode. The adhesion was identified in the boundary between the apical region of the regenerated heart and the surrounding tissue.

**Additional file 10: Video S8.** Representative echocardiography video of the injured heart that was repaired in a nonperfect regeneration manner 30 days after apical resection under B-mode. The adhesion was identified in the boundary between the apical region of the regenerated heart and the surrounding tissue.

**Additional file 11: Video S9.** Representative echocardiography video of the injured heart that was repaired in a nonperfect regeneration manner 45 days after apical resection under B-mode. The adhesion was identified in the boundary between the apical region of the regenerated heart and the surrounding tissue.

## Data Availability

All data generated or analysed during this study are included in this published article.

## Abbreviations

*X. tropicalis*: *Xenopus tropicalis*
daar: Days after apical resection
