# Supplementary figures and images for: Echocardiographic assessment of Xenopus tropicalis heart regeneration
Source: Cell Biosci. 2023 Feb 13;13:29. doi: 10.1186/s13578-023-00982-z (PMC9926761; doi:10.1186/s13578-023-00982-z)

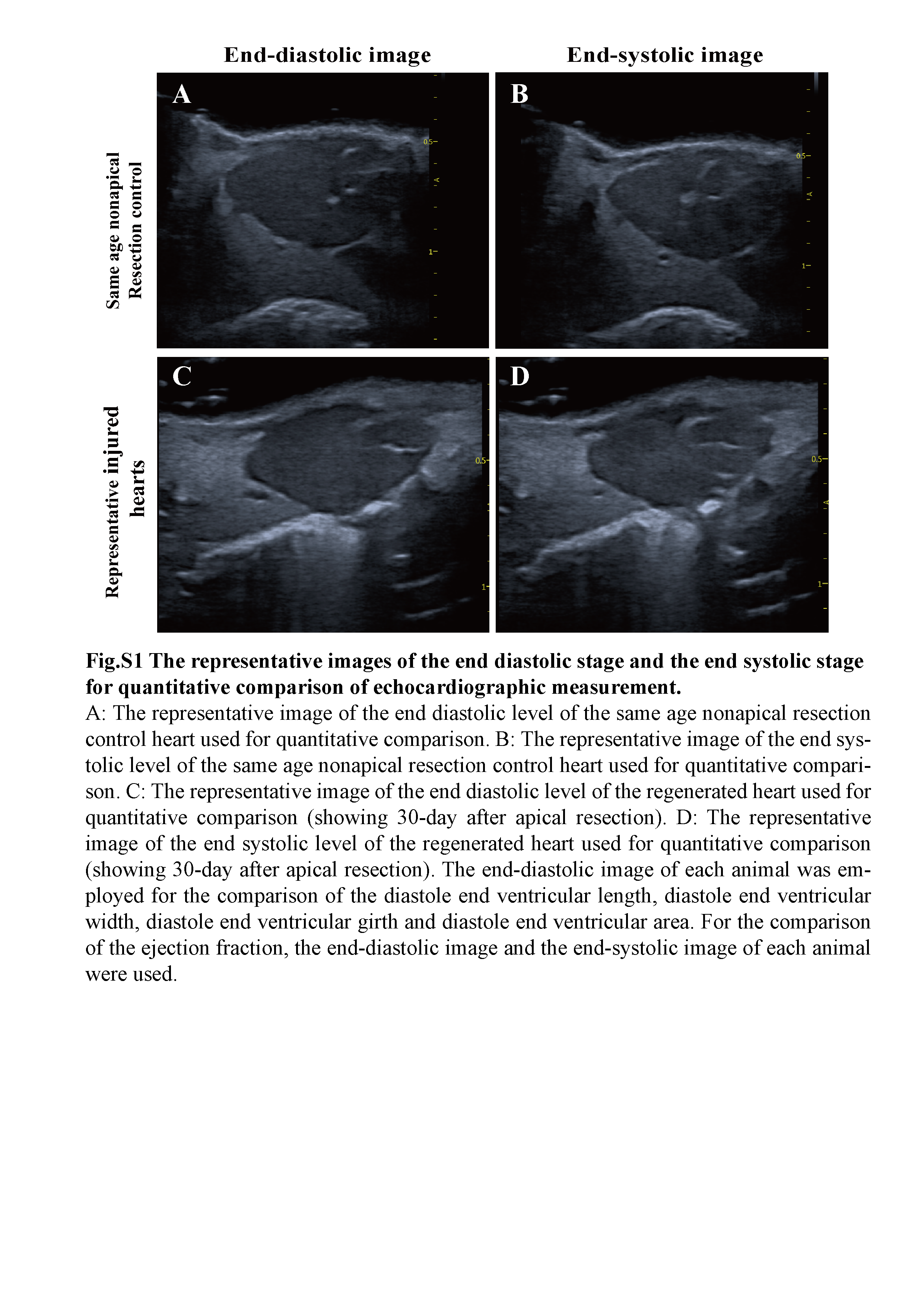

Supplement: Supplementary file 1 — Additional file 1: Figure S1. The representative images of the end diastolic stage and the end systolic stage for quantitative comparison of echocardiographic measurement. A: The representative image of the end diastolic level of the same age nonapical resection control heart used for quantitative comparison. B: The representative image of the end systolic level of the same age nonapical resection control heart used for quantitative comparison. C: The representative image of the end diastolic level of the regenerated heart used for quantitative comparison (showing 30 day after apical resection). D: The representative image of the end systolic level of the regenerated heart used for quantitative comparison (showing 30 day after apical resection). The end-diastolic image of each animal was employed for the comparison of the diastole end ventricular length, diastole end ventricular width, diastole end ventricular girth and diastole end ventricular area. For the comparison of the ejection fraction, the end-diastolic image and the end-systolic image of each animal were used. [file 13578_2023_982_MOESM1_ESM.png]
