# Supplementary material for: Echocardiographic assessment of Xenopus tropicalis heart regeneration
Source: Cell Biosci. 2023 Feb 13;13:29. doi: 10.1186/s13578-023-00982-z (PMC9926761; doi:10.1186/s13578-023-00982-z)
Supplement: Supplementary file 2 — Additional file 2: Table S1. Cardiac measurement data of young, young adult and adult X. tropicalis Heart using Echocardiography. [file 13578_2023_982_MOESM2_ESM.docx]

**Table S1: Cardiac measurement data of young, young adult and adult *X. tropicalis* Heart using Echocardiography.**

|  | **3M** | **6M** | **10M** | | **18M** | | **30M** | |
| --- | --- | --- | --- | --- | --- | --- | --- | --- |
|  | **(n=8)** | **(n=5)** | **female (n=3)** | **male (n=3)** | **female (n=9)** | **male (n=5)** | **female(n=5)** | **male(n=5)** |
| Body weight(g) | 2.84±0.61 | 6.58±0.85 ^#^ | 14.07±3.19 ^# *^ | 9.73±0.83 ^# *^ | 16.08±3.40 ^# *^ | 9.95±1.14 ^# *^ | 16.75±0.75 ^# *^ | 9.86±0.42 ^# *^ |
| Diastole end ventricular length (mm) | 3.49±0.52 | 3.91±0.29 | 5.79±0.30 ^# *^ | 5.01±0.60 ^# *^ | 5.76±0.58 ^# *^ | 5.30±0.35 ^# *^ | 5.96±0.57 ^# *^ | 5.53±0.25 ^# *^ |
| Diastole end ventricular width (mm) | 3.21±0.37 | 3.42±0.48 | 5.21±0.41 ^# *^ | 4.95±0.67 ^# *^ | 5.44±0.54 ^# *^ | 4.64±0.37 ^# * △^ | 5.90±0.33 ^# *^ | 4.85±0.43 ^# * △^ |
| Diastole end ventricular girth (mm) | 11.41±1.72 | 12.29±1.14 | 19.12±0.28 ^# *^ | 16.90±1.78 ^# *^ | 18.95±1.78 ^# *^ | 16.65±1.03 ^# * △^ | 20.29±1.51 ^# *^ | 18.00±1.04 ^# * △^ |
| Diastole end ventricular area (cm^2^) | 0.09±0.02 | 0.10±0.02 | 0.23±0.01 ^# *^ | 0.18±0.04 ^# * △^ | 0.24±0.04 ^# *^ | 0.18±0.03 ^# * △^ | 0.27±0.04 ^# *^ | 0.19±0.02 ^# * △^ |
| Ejection fraction (%) | 67.85±3.75 | 63.97±5.56 | 57.00±1.73 ^#^ | 59.84±5.95 ^#^ | 54.84±7.21 ^# *^ | 54.51±4.29 ^# *^ | 52.74±2.72 ^# *^ | 56.27±4.98 ^# *^ |
| Peak blood flow velocity (cm/s) | 8.95±1.75 | 12.67±2.22 | 9.48±2.96 | 9.11±1.80 | 13.57±5.48 ^#^ | 11.01±2.44 | 10.39±2.55 | 11.63±3.68 |
| Blood flow acceleration（cm/s^2^） | 71.90±8.61 | 72.61±17.59 | 111.63±15.59 | 130.52±12.90 ^#^ | 146.78±55.96 ^# *^ | 148.03±54.11 ^# *^ | 63.79±14.70 ^&^ | 56.18±6.86 ^&^ |
| Heart rate(times/min) | 62.50±4.81 | 56.40±3.13 ^#^ | 48.00±5.00 ^# *^ | 46.00±2.65 ^# *^ | 53.56±3.81 ^#^ | 47.60±5.68 ^# *^ | 46.80±5.17 ^# * &^ | 50.60±3.91 ^# *^ |

#: *p* < 0.05, vs. 3M. *: *p* < 0.05, vs. 6M. △: *p* < 0.05, vs. same age female. &: *p* < 0.05, vs. 18M (same gender and different gender comparison in H, comparison between the same gender in I).
